# Clinical Characteristics and Prognostic Factors in Patients with Gestational Trophoblastic Neoplasia: A Single-Center Study Comparing Ultra-High-Risk and Other Risk Groups

**DOI:** 10.3390/cancers17101655

**Published:** 2025-05-14

**Authors:** Atita Ruengsaen, Sethawat Sethasathien, Charuwan Tantipalakorn, Kittipat Charoenkwan, Prapaporn Suprasert, Jatupol Srisomboon, Theera Tongsong

**Affiliations:** Department of Obstetrics and Gynecology, Faculty of Medicine, Chiang Mai University, Chiang Mai 50200, Thailand; atitaning@gmail.com (A.R.); sethawat.s@cmu.ac.th (S.S.); kittipat.c@cmu.ac.th (K.C.); prapaporn.su@cmu.ac.th (P.S.); jatupol.s@cmu.ac.th (J.S.); theera.t@cmu.ac.th (T.T.)

**Keywords:** gestational trophoblastic neoplasia, ultra-high-risk, survival, prognostic factor

## Abstract

**Objective**: To evaluate treatment outcomes and prognostic factors in patients with ultra-high-risk gestational trophoblastic neoplasia (GTN) compared to those with low-risk and high-risk GTN. **Methods**: A retrospective review of medical records was conducted for GTN patients treated at Chiang Mai University Hospital, Chiang Mai, Thailand, between January 1999 and December 2019. Overall and risk-specific survival rates were estimated using the Kaplan–Meier method, and prognostic factors were analyzed through univariate and multivariate analyses. **Results**: During the study period, 160 patients with GTN were identified, including 98 (61.2%) classified as low-risk, 31 (19.4%) as high-risk, and 31 (19.4%) as ultra-high-risk. One patient in the low-risk group and one in the high-risk group underwent hysterectomy without adjuvant chemotherapy due to spontaneous regression of serum β-hCG (human chorionic gonadotropin). Additionally, one patient with ultra-high-risk GTN died before receiving chemotherapy. Among the 97 low-risk GTN patients, 80 (82.5%) were treated with either single-agent methotrexate or actinomycin D. Among the 30 high-risk GTN patients, 20 (66.7%) received EMA/CO (etoposide, methotrexate, actinomycin D, cyclophosphamide, and vincristine) as first-line chemotherapy, while 24 (80%) of the 30 ultra-high-risk GTN patients received EMA/CO as first-line treatment. Following first-line chemotherapy and/or salvage treatment, patients with ultra-high-risk GTN had significantly worse outcomes compared with those with low- and high-risk GTN, with remission rates of 63.3%, 96.9%, and 80.0%, respectively (*p* < 0.01). The five-year overall survival rate for patients with ultra-high-risk GTN was significantly lower than that for patients with low- and high-risk GTN (56% vs. 96% and 80%, respectively; *p* < 0.001). On multivariable analysis, significant prognostic factors included antecedent term pregnancy (hazard ratio [HR] = 11.50; 95% confidence interval [CI], 3.56–37.22; *p* < 0.01) and brain metastasis (HR = 4.61; 95% CI, 1.73–12.28; *p* < 0.01). **Conclusions**: Ultra-high-risk GTN accounts for only a small proportion of GTN cases but it is associated with poor survival rate and responsible for the majority of GTN-related deaths. Antecedent term pregnancy and brain metastasis were identified as significant prognostic factors.

## Simple Summary

This retrospective cohort study was conducted on 160 patients with gestational trophoblastic neoplasia (GTN), aimed to evaluate treatment outcomes and prognostic factors in patients with ultra-high-risk GTN compared with those with low-risk and high-risk GTN. The result highlights that ultra-high-risk GTN, defined as having a WHO score of ≥13, is associated with poor survival outcomes. Although it represents only a small proportion of GTN cases, it accounts for the majority of GTN-related deaths. The primary prognostic factors include a term antecedent pregnancy and the need for whole-brain radiotherapy. Salvage therapy may improve survival. Given the poor prognosis, ultra-high-risk patients should be recognized as a distinct subgroup requiring targeted research and specialized therapeutic strategies, including immunotherapy, targeted therapy, and induction chemotherapy.

## 1. Introduction

Gestational trophoblastic neoplasia (GTN) is a disease characterized by abnormal trophoblastic proliferation and can develop following any type of pregnancy [1,2]. The majority of antecedent pregnancies are hydatidiform moles [1,2]. The histopathological subtypes of GTN include invasive mole, choriocarcinoma, placental site trophoblastic tumor (PSTT), and epithelioid trophoblastic tumor (ETT) [1,2,3]. GTN following term pregnancy occurs in approximately 1 in 150,000 cases [2,4,5]. The precise prevalence of choriocarcinoma, PSTTs, and ETTs remains unclear, as these malignancies can arise after any type of pregnancy [6,7]. It varies geographically, with an estimated incidence of 1 in 40,000 pregnancies in North America and Europe, compared to a higher prevalence of 9.2 in 40,000 pregnancies in Southeast Asia [1]. GTN following a molar pregnancy is typically diagnosed during surveillance based on persistently elevated or rising serum β-hCG levels, often in the absence of symptoms. The clinical presentation of GTN depends on the extent of disease and metastatic involvement. Patients with non-metastatic GTN commonly present with abnormal vaginal bleeding, whereas those with lung metastases may experience dyspnea or hemoptysis, and those with brain metastases may develop neurological symptoms. GTN should be suspected in patients with unexplained abnormal bleeding or atypical clinical presentations, and serum β-hCG measurement should be included in the diagnostic evaluation.

The 2000 International Federation of Gynecology and Obstetrics (FIGO) staging and scoring system is used to classify the risk of patients diagnosed with gestational trophoblastic neoplasia (GTN), predict prognosis, and guide appropriate treatment [8]. This system was adapted from the WHO classification and reaffirmed in the 2021 update [9]. A 97% correlation of risk categorization was shown between the original WHO (1983) and FIGO 2000 systems [10]. GTN is a highly chemosensitive malignancy and it has the highest five-year survival rate (90.5%) among all gynecologic cancers [11]. In general, single-agent chemotherapy is the standard treatment for patients with low-risk GTN (FIGO score < 7), whereas multi-agent chemotherapy is recommended as the initial treatment for those with high-risk (FIGO score 7–12) and ultra-high-risk GTN (FIGO score ≥ 13) [3,12].

The prognosis for patients with low-risk GTN is excellent [13,14]. However, the survival rate for high-risk patients ranges from 70% to 90% [3,15,16,17,18], while that for ultra-high-risk patients is considerably lower, at approximately 62.5% to 68% [15,19]. Significant prognostic factors for GTN include disease stage, FIGO risk score, and the presence of liver or brain metastases [3].

Although several studies on GTN have been published, research specifically focusing on ultra-high-risk cases remains limited, with most studies including only a small number of patients, particularly when applying a score threshold of >13. For instance, even in the large series of 974 GTN patients reported by Bolze et al. [20], only 19 cases met the criteria for ultra-high-risk status. Furthermore, previous studies have used varying criteria to define ultra-high-risk cases. Earlier studies commonly applied a cutoff score of >12 [15,21], whereas the updated FIGO guidelines [3] and more recent studies [20,22] have established a score of >13. Therefore, a larger pooled dataset is needed in the literature to facilitate a more comprehensive meta-analysis, ensuring homogeneity in patient selection and enabling a robust evaluation of different treatment modalities. Given the variation in the natural course of the disease across geographical regions, its association with racial factors, and the limited number of studies on survival outcomes and prognostic factors in ultra-high-risk GTN in Southeast Asia, we conducted this study to evaluate the clinical characteristics, treatment outcomes, and prognostic factors in patients with ultra-high-risk GTN compared to those in the high- and low-risk groups. The findings from this study are expected to contribute to improving treatment strategies and enhancing survival outcomes in patients with GTN.

## 2. Materials and Methods

This study is a retrospective comparative analysis conducted using the gestational trophoblastic disease database of the Gynecologic Oncology Unit at Maharaj Nakorn Chiang Mai Hospital, Chiang Mai University, Thailand, from 1999 to 2019. The study was ethically approved by the institutional review board (The Research Ethics Committee 4, Faculty of Medicine, Chiang Mai University; Study Code: OBG-2563-07367).

**Database Development:** Data entry was performed by oncologic research assistants, and follow-up data were subsequently appended as appropriate. The recorded data included baseline patient characteristics, disease progression, risk scoring and staging, treatment modalities, clinical outcomes, and follow-up information.

**Data Retrieval:** The database was accessed by the authors to retrieve all patient records during the study period, which were then exported into the research dataset. Additionally, full medical records were reviewed comprehensively to validate essential data, including inclusion and exclusion criteria, scoring and staging, treatment details, chemotherapy regimens, clinical outcomes, complications, and health status at each follow-up visit. The exclusion criteria included patients with a placental-site trophoblastic tumor (PSTT) or epithelioid trophoblastic tumor (ETT), as well as those with incomplete medical records or follow-up data. The collected data for analysis included age, parity, pre- and post-treatment serum β-hCG levels, type of antecedent pregnancy, GTN histopathology, FIGO stage and risk score, interval from the index pregnancy, clinical signs and symptoms, tumor size, number of metastatic sites, imaging findings, prior failed chemotherapy, and types of interventions.

**Treatment:** The initial pretreatment evaluation comprised a detailed medical history, physical examination, transvaginal or transabdominal ultrasonography, chest radiography or computed tomography (CT), and routine laboratory investigations, including complete blood count, liver function tests, renal function tests, and serum β-hCG measurement. In cases where patients exhibited neurological symptoms, magnetic resonance imaging (MRI) or brain CT was additionally performed. Patients diagnosed with low-risk GTN received single-agent chemotherapy, either methotrexate (MTX) at a dose of 1 mg/kg (maximum 50 mg) administered intramuscularly on days 1, 3, 5, and 7, alternating with folinic acid at 0.1 mg/kg intramuscularly on days 2, 4, 6, and 8 (the MTX-FA regimen), or a 5-day intravenous actinomycin-D regimen at 0.5 mg/m^2^, repeated biweekly. Patients who exhibited resistance or failed to respond to single-agent chemotherapy were subsequently managed with multi-agent chemotherapy as salvage treatment.

Patients with high-risk and ultra-high-risk GTN received initial treatment with combination chemotherapy, including EMA/CO, EMA, EMA/EP (etoposide, cisplatin), or PI (cisplatin and ifosfamide) regimens, as deemed appropriate at the discretion of the attending physicians. Whole-brain radiation therapy at a total dose of 3000 cGy, delivered in 200-cGy daily fractions concurrent with chemotherapy, was administered to patients with brain metastases. Patients who failed EMA/CO were treated with salvage regimens, including EMA/EP, paclitaxel, and cisplatin alternating with paclitaxel and etoposide (TP/TE), or the ICE regimen (etoposide, ifosfamide, and cisplatin or carboplatin). After achieving normal serum β-hCG levels (<5 mIU/mL), an additional 2–4 courses of consolidation chemotherapy were administered, depending on the FIGO risk score.

**Post-Treatment Evaluation:** Following the completion of treatment, serum β-hCG levels were monitored weekly until normalization. Primary remission was defined as the normalization of serum β-hCG for three consecutive weeks, while complete remission was defined as sustained normal serum β-hCG levels for ≥6 months after achieving primary remission. Patients who did not attain primary remission with the first-line regimen were classified as chemo-resistant. Chemo-resistance was diagnosed if serum β-hCG levels declined by less than 10% over three consecutive measurements, increased over two consecutive measurements, or if new metastatic lesions were identified. After achieving complete remission, serum β-hCG levels were assessed monthly for 12 months, biannually until two years, and then annually for a minimum of five years.

**Statistical Analysis:** Data analysis was performed using the Statistical Package for the Social Sciences (SPSS), version 26.0 (IBM Corp., Armonk, NY, USA, Released 2019. IBM SPSS Statistics for Windows, Version 26.0: IBM Corp.). Continuous variables were summarized as either the median with interquartile ranges or the mean ± standard deviation (SD), depending on the distribution, which was assessed using the Kolmogorov–Smirnov test. Comparisons between groups for continuous variables were conducted using the Kruskal–Wallis test, while categorical variables were analyzed using the chi-squared test. Survival time was calculated from the date of diagnosis to the date of last follow-up or death from any cause, and overall survival rates were estimated using the Kaplan–Meier method. Univariable analysis to identify prognostic factors for survival was performed using the log-rank test. Multivariable analysis was subsequently conducted using Cox proportional hazard regression with a forward stepwise approach to evaluate the combined effects of risk factors identified in the univariate analysis. A two-sided *p*-value of <0.05 was considered indicative of statistical significance.

## 3. Results

**Clinical characteristics:** During the study period, 160 GTN patients were identified. Based on the FIGO risk score, 98 (61.2%), 31 (19.4%), and 31 (19.4%) patients were classified as having low-risk, high-risk, and ultra-high-risk GTN, respectively. The clinical characteristics of the patients are summarized in Table 1. The median ages of patients in the three groups were comparable: 35.5, 33.0, and 35.0 years, respectively. The median parity was not significantly different between groups. The most common presenting symptom was vaginal bleeding.

Clinical characteristics that were significantly more prevalent in the ultra-high-risk group compared to the low-risk and high-risk groups included elevated pretreatment serum β-hCG levels (*p* < 0.01), antecedent term pregnancy (*p* < 0.01), a prolonged interval from the index pregnancy (*p* < 0.01), hemoptysis (*p* = 0.01), hemiparesis (*p* = 0.01), histologic diagnosis of choriocarcinoma (*p* < 0.01), tumor size > 5 cm (*p* < 0.01), number and sites of metastases (*p* < 0.01), risk score (*p* < 0.01), and FIGO stage (*p* < 0.01). Among ultra-high-risk GTN patients, the median serum β-hCG level was 102,989 mIU/mL (range: 26,180–529,916 mIU/mL). Twenty (64.5%) patients in the ultra-high-risk group developed GTN following a term pregnancy, whereas GTN in the high-risk and low-risk groups most commonly followed a complete mole in 17 (54.8%) and 85 (86.7%) patients, respectively. Additionally, 22 (71.0%) patients in the ultra-high-risk group had an interval of more than 12 months between the antecedent pregnancy and the initiation of chemotherapy.

Eighty-seven (88.8%) patients in the low-risk group had FIGO stage I GTN, whereas 20 (64.5%) patients in the high-risk group had stage III disease, and 16 (51.6%) patients in the ultra-high-risk group had stage IV disease. Notably, two patients in the ultra-high-risk group were classified as stage I (one case) and stage II (one case). Among the 31 patients with ultra-high-risk GTN, 11 had brain metastases. Of these, 9 received whole-brain radiation therapy, and 5 underwent craniotomy: two for intracranial hemorrhage and three for brain herniation.

**Treatment outcomes:** Among the 160 patients, one patient in the low-risk group and one in the high-risk group underwent hysterectomy without adjuvant chemotherapy due to spontaneous regression of serum β-hCG levels. One patient with ultra-high-risk GTN died before receiving chemotherapy. Consequently, these three cases were excluded from the analysis of treatment outcomes.

The median follow-up times were 91 months (range: 48–118 months) for low-risk patients, 116 months (range: 42–137 months) for high-risk patients, and 30 months (range: 12–96 months) for ultra-high-risk patients. Table 2 summarizes the outcomes after treatment with first-line, second-line, and salvage chemotherapy for GTN patients in the three groups.

Among the 97 patients in the low-risk group, 68 (70.1%) were treated with the MTX-FA regimen, 12 (12.4%) with the 5-day actinomycin-D regimen, and 17 (17.5%) with the weekly MTX regimen as first-line chemotherapy. Of these 97 low-risk GTN patients, 66 (68.0%) achieved complete remission after first-line treatment. The remaining 31 patients who failed first-line chemotherapy were treated with second-line and subsequent salvage chemotherapy, resulting in an overall cure rate of 96.9% in the 97 patients with low-risk GTN, as shown in Table 2. In the 30 patients with high-risk GTN, 20 were treated with the EMA/CO regimen and 10 with the EMA regimen as first-line chemotherapy, resulting in a complete remission rate of 66.7%. The 10 patients who failed first-line treatment received second-line and subsequent salvage chemotherapy, yielding an overall cure rate of 80.0% in the 30 patients with high-risk GTN, as shown in Table 2.

Among the 30 patients with ultra-high-risk GTN, 24 (80%) received the EMA/CO regimen as first-line chemotherapy, with 15 (62.5%) achieving complete remission. The remaining six patients were treated with the EMA regimen (five patients) and the EMA/EP regimen (one patient) as first-line chemotherapy. The complete remission rate after first-line treatment was 63.3%. Following subsequent second-line and salvage chemotherapy, an overall cure rate of 63.3% was achieved in the 30 patients with ultra-high-risk GTN, as shown in Table 2.

**Figure 1:**
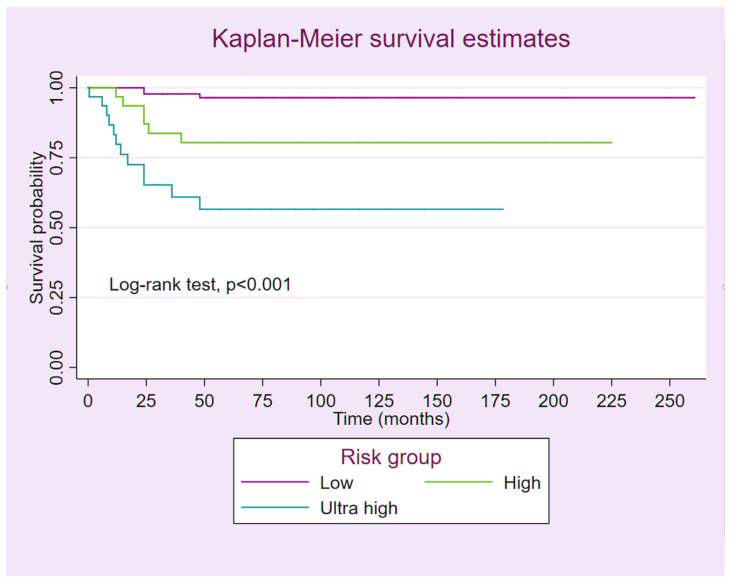
The estimate of 5-year overall survival rate of the GTN patients among the 3 FIGO risk groups.

**Prognosis:** Among the 157 GTN patients, 20 died from the disease. Three of the ninety-seven patients in the low-risk group died from respiratory failure due to lung metastasis. Six of the thirty patients in the high-risk group died from respiratory failure caused by lung metastasis (five patients) and brain metastasis (one patient). Of the 30 patients with ultra-high-risk GTN, 7 died from brain metastasis and 4 died from respiratory failure due to lung metastasis. Figure 1 shows the estimates of the 5-year overall survival rate for patients in the three risk groups, as depicted by Kaplan–Meier curves. Patients with ultra-high-risk GTN had a significantly lower survival rate compared to those with low-risk and high-risk GTN (56% vs. 96% and 80%, respectively; log-rank test: *p* < 0.001).

The prognostic factors are summarized in Table 3. Univariate analysis revealed that non-molar antecedent pregnancy, as well as brain and spleen metastases, had a significant impact on the survival rate. However, multivariate analysis identified only antecedent term pregnancy and brain metastasis as significant prognostic factors (*p* < 0.01).

## 4. Discussion

The main findings of this study are as follows. Ultra-high-risk GTN accounts for only a small proportion of GTN cases but it is responsible for the majority of GTN-related deaths. Patients with ultra-high-risk GTN had a relatively low survival rate of 56%, which is lower than the rates reported in most Western studies [23]. Additionally, ultra-high-risk GTN is predominantly associated with antecedent remote-term pregnancy. Notably, this study contributes a substantial number of ultra-high-risk cases to the literature, providing valuable data for future meta-analyses.

Among the 160 GTN patients in our study, 31 (19.4%), or approximately one-fifth, were classified as the ultra-high-risk group. Compared to those in the low-risk and high-risk groups, patients with ultra-high-risk GTN had significantly higher scores of the WHO prognostic factors, including elevated serum β-hCG levels, antecedent term pregnancy, a prolonged interval from the index pregnancy, histologic diagnosis of choriocarcinoma, tumor size >5 cm, and a greater number and distribution of metastases.

Not surprisingly, despite initiating treatment with multi-agent chemotherapy combined with surgical intervention and adjuvant radiation, the remission rate among patients with ultra-high-risk GTN in our study was only 63.3%, significantly lower than that observed in the low-risk (96.9%) and high-risk (80%) groups. Similarly, patients with ultra-high-risk GTN had a significantly lower survival rate compared to those with low-risk and high-risk GTN (56% vs. 96% and 80%, respectively). The survival outcomes of GTN patients in this study were comparable, though relatively lower, to those reported in previous studies [15,17,19,20,24,25]. Likewise, the remission rate of ultra-high-risk GTN patients in our study aligned with the previously reported range of 62–68% [15,19,20,26].

The most commonly used first-line chemotherapeutic regimen in this study was EMA/CO, which accounted for 80% of cases and achieved a remission rate of 68.2%. Similarly, Bolze et al. [20] reported a 5-year survival rate of 61.6% in a series of 11 ultra-high-risk patients in France who received EMA/CO as a first-line regimen. However, the optimal treatment regimen for ultra-high-risk GTN remains unclear. A study from Thailand reported a 5-year survival rate of 62.5% (5 out of 8 patients) using VAC (vincristine, actinomycin-D, and cyclophosphamide) as the first-line chemotherapy and EMA/CO as the second-line regimen [19]. Additionally, Kong et al. [15] reported a 5-year overall survival rate of 67.9% in 143 ultra-high-risk patients in China who were treated with FAVE (floxuridine, actinomycin-D, etoposide, and vincristine) as the first-line regimen. This study contributes additional data on EMA/CO as a first-line chemotherapy regimen, which may be valuable for future meta-analyses aimed at identifying the most effective treatment for ultra-high-risk patients. However, data on second-line regimens, including our findings, remain heterogeneous, with small sample sizes limiting definitive conclusions. Larger studies are needed to enable more robust analyses. A recent meta-analysis by Albright et al. [23] on the treatment of high-risk GTN (WHO score > 10) reported an 80% complete response rate to primary treatment with EMA/CO or EMA/EP, with a low relapse risk of <5% following complete response. Notably, the mortality rate among ultra-high-risk patients was 7.4 times higher than that of high-risk patients, underscoring the urgent need to explore novel first-line regimens for this population.

It is important to note that risk factors have been reported inconsistently across various studies. For example, while many studies have identified brain and lung metastases as prognostic factors for GTN [3,11], some have found that lung metastases are not significantly associated with prognosis. This discrepancy is likely due to the small sample sizes, given the rarity of the disease, which may limit statistical power and prevent the detection of significant associations when the effect size is modest. Nevertheless, most studies, including our own, consistently indicate that brain metastasis is an independent prognostic factor. Our findings demonstrated a fourfold increase in mortality risk in the presence of brain metastases. Similarly, Kong et al. [15] reported that brain metastases increased the risk of ultra-high-risk GTN by 2.2 times. Moreover, brain metastases remain a strong predictor of progression to more severe forms of GTN. In comparison, Jiang et al. [27] highlighted that the presence of more than eight metastatic sites substantially increases the risk, with an approximately eightfold greater likelihood of GTN occurrence.

Antecedent pregnancy is another prognostic factor, with term pregnancy associated with a 14-fold higher risk of GTN compared to complete mole. Similarly, abortion carries an approximately fivefold greater risk than complete mole. This prognostic factor is consistent with findings from other studies. For example, term pregnancy and abortion have been reported to increase the risk of GTN occurrence by 2.2 and 2.7 times, respectively, compared to molar pregnancy. Notably, term pregnancy has been recognized as a significant prognostic factor for GTN for over 30 years [28,29].

Increasing age is associated with a higher risk of developing GTN [30], a finding consistent with our study. Each additional year of age increases the likelihood of more severe GTN by a factor of 1.04. Older women are also more likely to experience abnormal fertilization [31].

**Research Implications:** A larger cohort of ultra-high-risk GTN patients receiving various treatment modalities is still needed to accumulate in literature and facilitate future analyses with more reliable results. Additionally, ultra-high-risk GTN should be consistently defined using standardized criteria, specifically a FIGO score >13, to ensure greater homogeneity in the study of this rare condition. Given the poor survival rate, further research on emerging therapeutic approaches, such as immunotherapy, targeted or personalized therapy, and induction chemotherapy, is warranted for this high-risk population.

**Limitations and strength:** The main limitation is primarily due to its retrospective nature. Missing data were a concern, particularly regarding detailed information on chemotherapy toxicity. Despite two decades of case collection, the number of patients in the high-risk and ultra-high-risk groups remained relatively small. Additionally, various chemotherapy regimens were used for treatment, both as first-line and salvage therapy, even among patients classified within the same risk group. A key strength of our study is that all patients were treated at a single institution, ensuring consistent treatment planning and surveillance.

## 5. Conclusions

In conclusion, ultra-high-risk GTN is associated with poor survival outcomes. Although it represents only a small proportion of GTN cases, it accounts for the majority of GTN-related deaths. The primary prognostic factors include a term antecedent pregnancy and the need for whole-brain radiotherapy. Salvage therapy may improve survival. Given the poor prognosis, ultra-high-risk patients should be recognized as a distinct subgroup requiring targeted research and specialized therapeutic strategies, including immunotherapy, targeted therapy, and induction chemotherapy.

## Figures and Tables

**Table 1:** Comparisons of clinical characteristics of the patients among the three risk groups.

		Risk Group		
Characteristics	Low (n = 98)	High (n = 31)	Ultra-High (n = 31)	*p*-Value
Age (yrs)	35.50 (27.0–46.0)	33.0 (30.0–44.0)	35.0 (27.0–49.0)	0.89
Parity	1.00 (0.00–2.00)	1.00 (0.00–2.00)	2.00 (1.00–3.00)	0.07
Pretreatment serum β-hCG (mIU/mL)	2897 (441–18,591)	82,476 (16,000–171,800)	102,989 (26,180–529,916)	<0.01
Antecedent pregnancy				<0.01
• Complete mole	85 (86.7)	17 (54.8)	5 (16.1)	
• Partial mole	11 (11.2)	0 (0.0)	1 (3.2)	
• Abortion	1 (1.0)	7 (22.6)	5 (16.1)	
Term	1 (1.0)	7 (22.6)	20 (64.5)	
Interval from index pregnancy (months)				<0.01
• <4	87 (88.8)	15 (48.4)	4 (12.9)	
• 4–6	4 (4.1)	1 (3.2)	2 (6.5)	
• 7–12	2 (2.0)	2 (6.5)	3 (9.7)	
• >12	5 (5.1)	13 (41.9)	22 (71.0)	
Sign and symptom *				
• Vaginal bleeding	92 (93.9)	22 (71.0)	17 (54.8)	<0.01
• Pelvic mass	9 (9.2)	6 (19.4)	6 (19.4)	0.14
• Pelvic pain	8 (8.2)	4 (12.9)	5 (16.1)	0.31
• Hemoptysis	0 (0.0)	3 (9.7)	6 (19.4)	0.01
• Hemiparesis	0 (0.0)	0 (0.0)	4 (12.9)	0.01
• Others	0 (0.0)	1 (3.2)	5 (16.1)	<0.01
Histologic diagnosis				<0.01
• Invasive mole	9 (9.2)	1 (3.2)	2 (6.5)	
• Choriocarcinoma	7 (7.1)	16 (51.6)	23 (74.2)	
• No tissue for pathologic exam	82 (83.7)	14 (45.2)	6 (19.4)	
• Largest tumor size (cm.)				<0.01
• <3	81 (82.7)	10 (32.3)	2 (6.5)	
• 3–5	9 (9.2)	3 (9.7)	4 (12.9)	
• >5	8 (8.2)	18 (58.1)	25 (80.6)	
Sites of metastasis				
• Lung	11 (11.2)	21 (67.7)	25 (80.6)	<0.01
• Vaginal	0 (0.0)	1 (3.2)	3 (9.7)	0.01
• Pelvic organ	1 (1.0)	3 (9.7)	1 (3.2)	0.05
• Liver	0 (0.0)	1 (3.2)	6 (19.4)	<0.01
• Brain	0 (0.0)	0 (0.0)	11 (35.5)	<0.01
• Renal	0 (0.0)	0 (0.0)	2 (6.5)	0.38
• Spleen	0 (0.0)	0 (0.0)	3 (9.7)	0.07
• Bone	0 (0.0)	0 (0.0)	2 (6.5)	0.38
• Pancreas	0 (0.0)	0 (0.0)	1 (3.2)	0.38
Number of metastases				<0.01
• 0	87 (88.8)	7 (22.6)	1 (3.2)	
• 1–4	9 (9.2)	7 (22.6)	5 (16.1)	
• 5–7	0 (0.0)	6 (19.4)	3 (9.7)	
• >8	2 (2.0)	11 (35.5)	22 (71.0)	
Risk score	2 (1–4)	9 (8–11)	14 (13–16)	<0.01
FIGO stage				<0.01
• I	87 (88.8)	8 (25.8)	1 (3.2)	
• II	1 (1.0)	2 (6.5)	1 (3.2)	
• III	10 (10.2)	20 (64.5)	13 (41.9)	
• IV	0 (0.0)	1 (3.2)	16 (51.6)	
Previous failed chemotherapy				0.16
• No	86 (87.8)	26 (83.9)	25 (80.6)	
• Single drug	12 (12.2)	4 (12.9)	4 (12.9)	
• Two drugs	0 (0.0)	1 (3.2)	2 (6.5)	

* The patients may have multiple symptoms. Data are expressed by median (interquartile range) and number (percentage). The Kruskal–Wallis test was used to compare between groups among continuous variables. The Fisher’s exact test was used to compare between groups among categorical variables.

**Table 2:** Response after first-line, second-line, and salvage chemotherapy in 157 GTN patients.

Characteristics	Low (n = 97)	High (n = 30)	Ultra-High (n = 30)	*p*-Value
Response after first line				0.91
• Remission	66 (68.0)	20 (66.7)	19 (63.3)	
• Resistant	31 (32.0)	10 (33.3)	11 (36.7)	
Response after second line				0.04
• Remission	19 (61.3)	4 (40.0)	2 (18.2)	
• Resistant	12 (38.7)	6 (60.0)	9 (81.8)	
Response after salvage therapy				<0.01
• Remission	11 (91.7)	1 (16.7)	2 (22.2)	
• Resistant	1 (8.3)	5 (83.3)	7 (77.8)	
Vital status				<0.01
• Alive	94 (96.9)	24 (80.0)	19 (63.3)	
• Died of disease	3 (3.1)	6 (20.0)	11 (36.7)	

Data are expressed by number (percentage). Fisher’s exact test was used to compare between groups.

**Table 3:** Cox’s proportional hazard regression of prognostic factors.

	Univariable Analysis		Multivariable Analysis	
Variables	HR (95% CI)	*p*-Value	aHR (95% CI)	*p*-Value
Age (years)	1.02 (0.98–1.06)	0.27		
Antecedent pregnancy		<0.01		<0.01
• Complete mole	1		1	
• Partial mole	2.21 (0.25–19.80)		2.31 (0.26–20.69)	
• Abortion	6.46 (1.45–28.86)		4.02 (0.84–19.22)	
• Term	15.99 (5.20–49.14)		11.50 (3.56–37.22)	
Pretreatment β-hCG (mIU/mL)		0.72		
• <100,000	1			
• ≥100,000	1.20 (0.44–3.27)			
Previous failed chemotherapy		0.51		
• No	1			
• Single drug	1.19 (0.35–4.06)			
• Two drugs	3.27 (0.44–24.62)			
Liver metastasis		0.24		
• No	1			
• Yes	2.39 (0.56–10.25)			
Brain metastasis		<0.01		<0.01
• No	1		1	
• Yes	11.43 (4.59–28.48)		4.61 (1.73–12.28)	
Renal metastasis		0.07		
• No	1			
• Yes	6.58 (0.88–49.10)			
Spleen metastasis		<0.01		
• No	1			
• Yes	12.93 (2.99–55.85)			

Abbreviation: HR = hazard ratio, aHR = adjusted hazard ratio.

## Data Availability

The datasets analyzed during the current study are available from the corresponding author upon reasonable request.
